# Efficacy of ultrasonography and Tc-99m MIBI SPECT/CT in preoperative localization of parathyroid adenomas causing primary hyperthyroidism

**DOI:** 10.1186/s12880-021-00616-1

**Published:** 2021-05-21

**Authors:** Ruigang Lu, Wei Zhao, Li Yin, Ruijun Guo, Bojun Wei, Mulan Jin, Xiang Zhou, Chun Zhang, Xiuzhang Lv

**Affiliations:** 1 Department of Ultrasound, Beijing Chaoyang Hospital, Capital Medical University, No. 8 Gongren Tiyuchang Nanlu, Chao Yang District, Beijing, 100020 China; 2 Department of Thyroid and Neck Surgery, Beijing Chaoyang Hospital, Capital Medical University, No. 8 Gongren Tiyuchang Nanlu, Chao Yang District, Beijing, 100020 China; 3 Department of Pathology, Beijing Chaoyang Hospital, Capital Medical University, No. 8 Gongren Tiyuchang Nanlu, Chao Yang District, Beijing, 100020 China; 4 Department of Radiology and Nuclear Medicine, Xuanwu Hospital, Capital Medical University, No. 45 Changchun Street, Xicheng District, Beijing, 100053 China; 5 Department of Echocardiography, Beijing Chaoyang Hospital, Capital Medical University, No. 8 Gongren Tiyuchang Nanlu, Chao Yang District, Beijing, 100020 China

**Keywords:** Ultrasonography, Tc-99m MIBI SPECT/CT, Primary hyperparathyroidism, Parathyroid adenoma

## Abstract

**Background:**

Primary hyperparathyroidism (PHPT) results from an excess of parathyroid hormone (PTH) produced from an overactive parathyroid gland. The study aimed to explore the sonographic features of parathyroid adenomas and assess the diagnostic performance of ultrasonography (US) and Tc-99m MIBI SPECT/CT for preoperative localization of parathyroid adenomas.

**Methods:**

A total of 107 patients were enrolled in this retrospective study who had PHPT and underwent parathyroidectomy. Of the 107 patients, 97 performed US and Tc-99m MIBI SPECT/CT examinations for preoperative localization of parathyroid nodules. The sensitivity and accuracy of each modality were calculated.

**Results:**

In this study, residual parathyroid sign and polar vascular sign were identified as characteristic US features of parathyroid adenomas. These manifestations were closely related to the size of the abnormal parathyroid lesions. Among the 108 parathyroid nodules from 97 patients with PHPT, the sensitivity and accuracy of US for locating the parathyroid nodules were significantly higher than those of Tc-99m MIBI SPECT/CT (93.0% vs. 63.0% and 88.0% vs. 63.0% respectively; ^2^=26.224, 18.227 respectively, P<0.001). The differences between US+Tc-99m MIBI SPECT/CT and Tc-99m MIBI SPECT/CT-alone were statistically significant (^2^=33.410, 21.587 respectively, P<0.001), yet there were no significant differences in the sensitivity or accuracy between US+Tc-99m MIBI SPECT/CT and US-alone (^2^=0.866, 0.187 respectively, P=0.352 and 0.665).

**Conclusions:**

US shows significantly better sensitivity and accuracy for localization of parathyroid adenomas than Tc-99m MIBI SPECT/CT. However, US combined with Tc-99m MIBI SPECT/CT is of great clinical value in the preoperative localization of parathyroid nodules in patients with PHPT.

## Background

Primary hyperparathyroidism (PHPT) represents one of the most common endocrine disease, usually results from an excess of parathyroid hormone (PTH) produced from an overactive parathyroid gland. In the general population, the incidence of PHPT is approximately 0.10.7% [1]. The prevalence of PHPT is affected by age, sex, race, and gender, with postmenopausal women showing the highest prevalence worldwide. In recent years, its prevalence has increased further worldwide, which has been attributed, in part, to improved screening methodologies, usage of lithium and thiazide drugs, and increased rates of obesity and hypertension [2,4]. In Europe and the United States, PHPT is considered to be the third most common endocrine disease after diabetes and hyperthyroidism [5].

The diagnosis of PHPT is based upon elevated levels of both blood serum calcium and PTH, after excluding other causes of hyperparathyroidism or hypercalcemia. The classical manifestations of PHPT include musculoskeletal and renal disorders, such as kidney stones and nephrocalcinosis, along with cardiovascular, neuromuscular, and gastrointestinal symptoms. In patients with PHPT, adenomas are the most common and account for 7389% of cases, while hyperplasia (1121%) and parathyroid carcinomas (0.55.0%) are less common [6]. In terms of surgical approaches, recent studies have shown similar cure rates between minimally invasive parathyroidectomy and bilateral neck exploration [7, 8]. However, the success of minimally invasive surgery is highly dependent on the accurate preoperative detection and localization of abnormal parathyroid lesions.

The two most widely utilized screen modalities for the preoperative detection and localization of parathyroid adenomas are ultrasonography (US) and Tc-99m sestamibi (MIBI) single-photon emission computed tomography/computed tomography (SPECT/CT). Exploring characteristics of US in patients with PHPT is of great significance for the accurate detection and localization of parathyroid adenomas.

## Methods

### Patient selection and study approval

In total, 107 patients consecutively diagnosed with PHPT in our institution between May 2018 and July 2020 were enrolled in this study. There were three exclusion criteria, including PHPT caused by post-operative incision implantation, secondary hyperparathyroidism, and tertiary hyperparathyroidism due to chronic renal disease. This study protocol was approved by the Institutional Review Board of Beijing Chaoyang Hospital, Capital Medical University. Due to the retrospective nature of this study, informed consent was waived by the committee.

### US imaging

A senior radiologist (Dr. Lu Ruigang), with more than 16years of experience, evaluated the US images. US was performed using a 514Hz probe on the Canon Aplio 500 system (Canon Medical, Inc.). For scanning, the patients head was tilted to the side. The scanning area extended up to the mandible, down to the supraclavicular fossa, and on both sides to the lateral edge of the sternocleidomastoid muscle. The key scanning areas included the dorsal and inferior sides of the inferior pole of the thyroid, along with the dorsal side of the middle and upper part of the thyroid. The location, size, boundary, echo, calcification or cyst, and blood supply of the nodules were recorded.

### SPECT/CT imaging

Following an intravenous injection of 555MBq ^99m^Tc-MIBI, early (10min) and late (120min) static images of 10min of the neck and mediastinum were obtained using a dual-head combined SPECT/CT camera (Infinia Hawkeye 4, GE Healthcare, USA) with a low-energy high-resolution collimator in a 128128 matrix. SPECT/CT was performed at the 120th minute immediately after the static images of the neck and mediastinum were obtained. SPECT data were acquired over 360, yielding 60 projections at 15s per projection in a 128128 matrix with a 1.0 zoom. Computed tomography (CT) parameters were a tube current of 2.5 mAs, a voltage of 140 kVp, and 5mm slice thickness.

A nuclear medicine specialist (Dr. Zhang Chun) with more than 20years of experience assessed the Tc-99m MIBI SPECT/CT examinations, who was blinded to all clinical, radiological, and laboratory data. The ^99m^Tc-MIBI SPECT/CT study was considered positive if focal activity retention was detectable on both early and late static images. Increased focal uptake areas were identified by SPECT, but not on planar images, and with a corresponding asymmetrical nodular lesions on CT were also interpreted as a positive scan. When the resected lesion found by US or Tc-99m MIBI SPECT/CT was confirmed as a parathyroidal abnormality pathologically, the lesion was defined as positive on US or Tc-99m MIBI SPECT/CT.

### Statistical analysis

SPSS 23.0 software (IBM, Chicago, IL, USA) was used for statistical analysis. Measurement data were expressed as meanstandard deviation and receiver operating characteristic (ROC) curves of working characteristics were drawn. Numerical data were expressed as percentages, and comparisons between groups were conducted by the Pearsons chi-square tests. When the difference was statistically significant, row x column partition (subdividing RxC table) was used for pairwise comparison. P-values<0.05 were considered statistically significant.

## Results

There were 120 nodules in 107 patients (76 females and 31 males, aged 1374) including 70 cases of single parathyroid adenoma, three cases of double adenoma (six nodules), one case of multiple endocrine neoplasia type 1 (MEN1) with four nodules, and one case of atypical parathyroid adenoma, twelve cases of ectopic parathyroid adenoma, one case of parathyroid lipoadenoma, four cases of parathyroid carcinoma, six cases of parathyroid hyperplasia with 13 nodules, three cases of parathyroid cysts, one case of a normal parathyroid gland, two cases of thyroid follicular adenoma, one case of papillary carcinoma, one case of nodular goiter, and one case of cervical lymph node (Table 1). The maximum diameter of the parathyroid nodules ranged from 0.35 to 5.80cm (including nodules with maximum diameter 1.0cm in 104 cases and<1.0cm in 16 cases).

**Table 1:** Patient demographics and baseline characteristics

Parameter	Value
*Mean age (years)*	54 (1376)
Gender	
Women n, (%)	76 (71.0%)
Men n, (%)	31 (29.0%)
*Pathological findings*	
Parathyroid single adenoma	70
Parathyroid double adenomas	3 (6 nodules)
Multiple endocrine neoplasia type 1	1 (4 nodules)
Atypical adenoma	1
Ectopic parathyroid adenoma	12
Lipoadenoma	1
Parathyroid carcinoma	4
Primary parathyroid hyperplasia	6 (13 nodules)
Parathyroid cysts	3
Parathyroid	1
Thyroid follicular adenoma	2
Papillary thyroid microcarcinoma	1
Nodular goiter	1
Lymph nodes	1

**Fig. 1:**
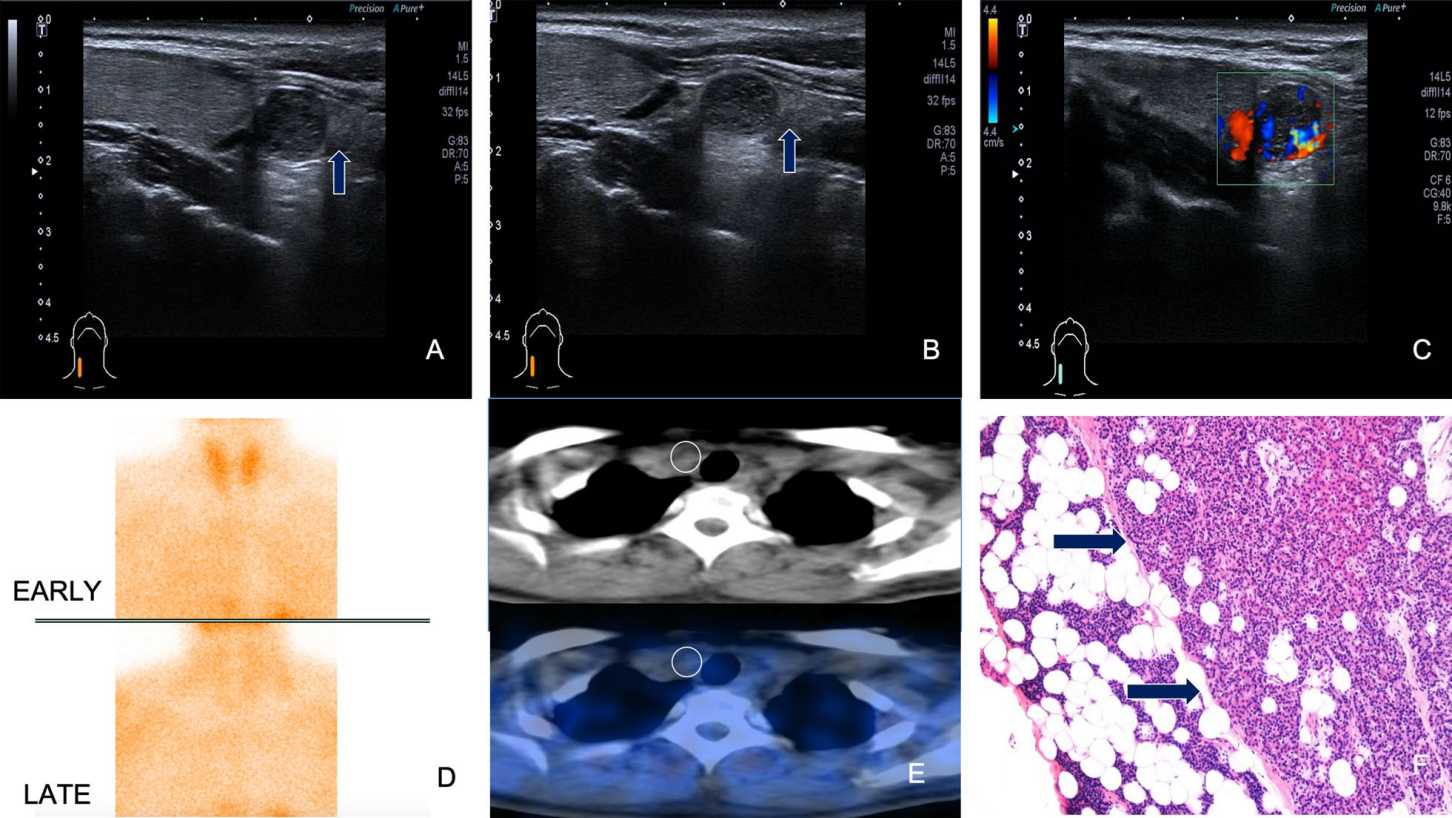
Parathyroid adenoma below the inferior pole of the right lobe of thyroid. The right inferior parathyroid adenoma clearly represents the residual parathyroid sign. Nodule and the thyroid gland move relatively with swallowing (**a**, **b**). Abundant blood flow signals on CDFI (**c**). The nodule was negative on planar images and Tc-99m MIBI SPECT/CT (**d**, **e**). The pathological results showed parathyroid adenoma (mainly chief cells and eosinophils), and fibrous capsule formation could be seen with the surrounding normal parathyroid gland (**f**)

**Fig. 2:**
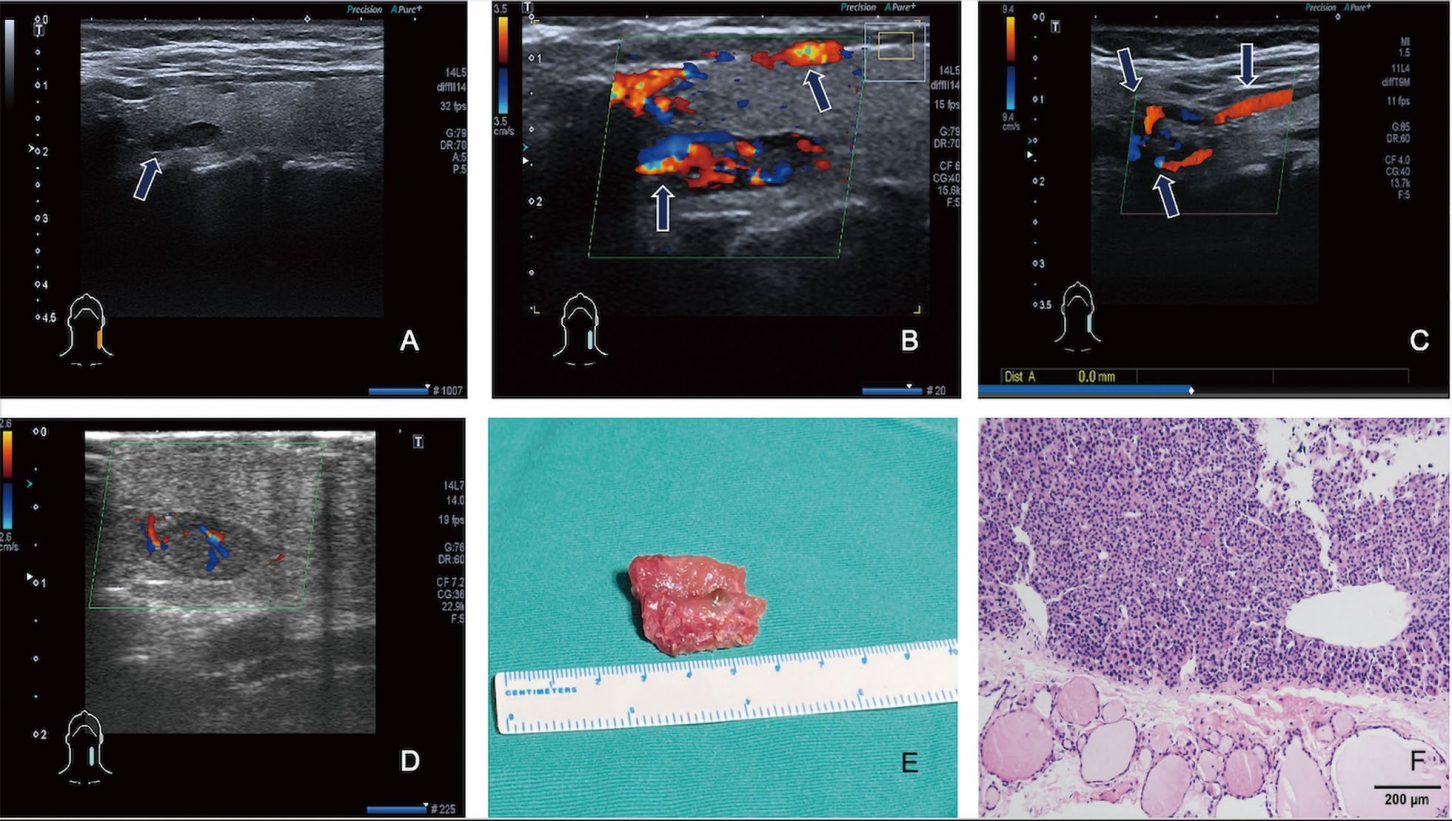
A complete type of intrathyroid parathyroid adenoma in the left lobe of thyroid. A homogeneously hypoechoic nodule in the left lobe of thyroid (**a**) that was negative in Tc-99m MIBI SPECT/CT, and the polar vascular sign (the supply vessel from the posterior branch of the superior thyroid artery) and anterior branch were detected by US (**b**, **c**). After clipping the superior thyroid artery intraoperatively, the blood supply in the nodule decreased significantly, which confirmed that the blood supply of the nodule came from the superior thyroid artery (**d**). After partial thyroidectomy and the nodule was resected, the US showed the parathyroid nodule was completely removed (**e**). The pathological results showed the parathyroid adenoma and surrounding thyroid tissue (**f**)

**Fig. 3:**
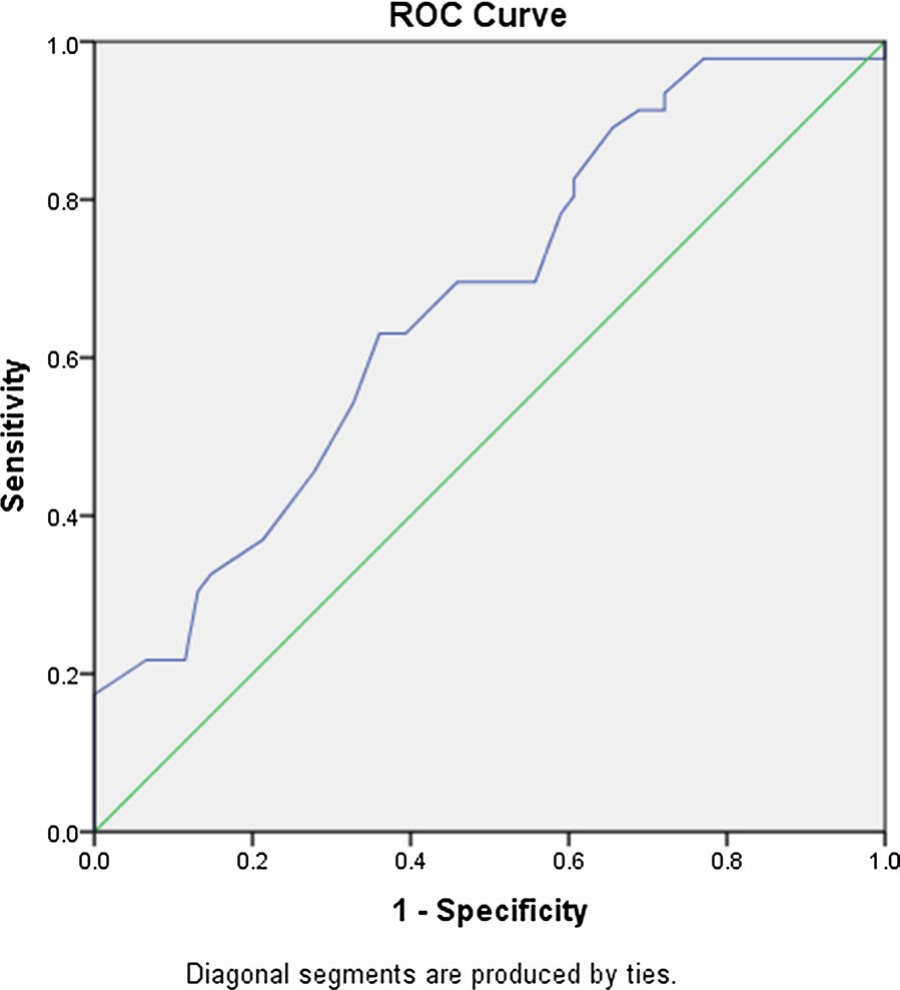
Receiver-operating characteristics (ROC) curve analysis for the residual parathyroid sign. When the maximum diameter of the nodule was 1.55cm, the sensitivity and specificity of the residual parathyroid sign in predicting parathyroid adenoma were 63.0% and 63.9%, respectively

**Fig. 4:**
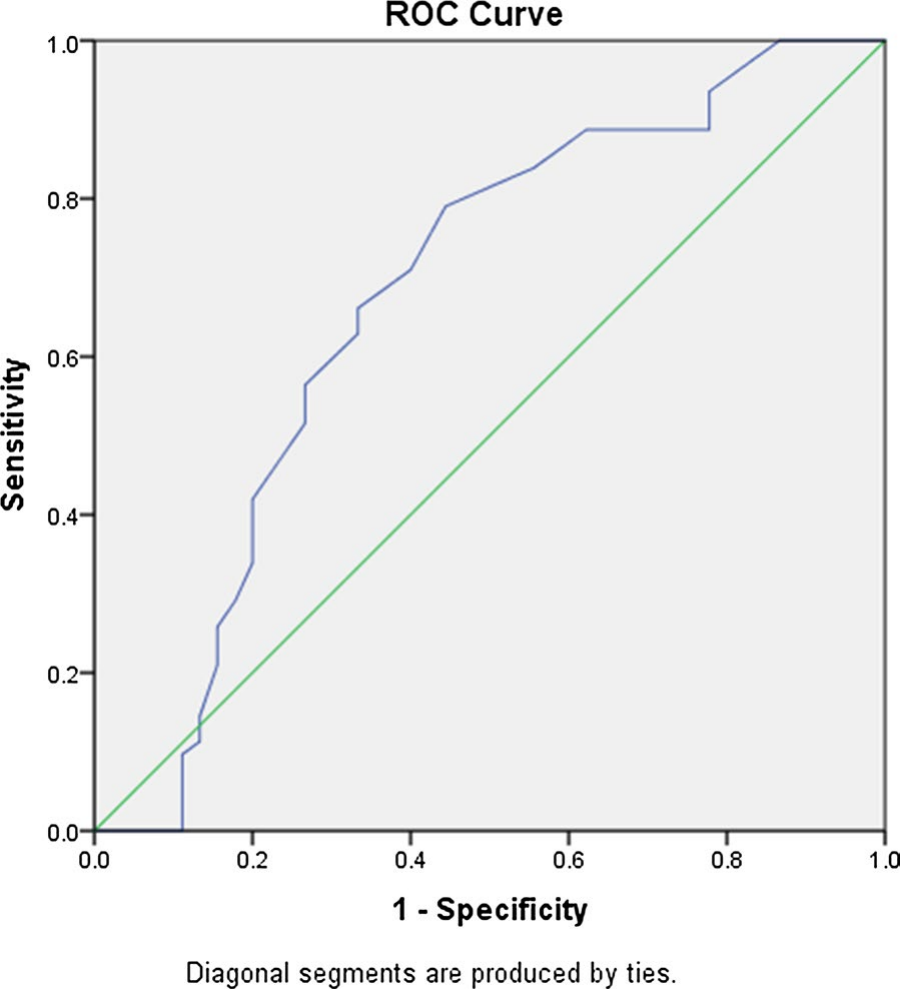
Receiver-operating characteristics (ROC) curve analysis for the polar vascularity sign. When the maximum diameter of the nodule was 1.35cm, the sensitivity and specificity of the residual parathyroid sign in predicting parathyroid adenoma were 79.0% and 55.6%, respectively

Parathyroid adenomas appeared as homogeneously hypoechoic nodules under US, with echogenic thyroid capsule separating them from the thyroid tissues, along with abundant blood flow. In addition, residual parathyroid signs and polar vascular signs are confirmed as characteristic sonographic features of parathyroid adenomas and these manifestations are closely related to the size of the parathyroid abnormal lesions (Figs.1, 2). In total, 46 of the 107 parathyroid nodules (of the 120 nodules, there were six cases of non-parathyroid lesions and seven cases of false negative on US, which were excluded) had residual parathyroid signs. The AUC for predicting residual parathyroid signs by the maximum diameter of parathyroid nodules was 0.670, significantly different from that of 0.05 (P=0.005). However, the accuracy of using the maximum diameter of parathyroid nodules to predict parathyroid sign was low (0.5<Az0.7), with a Youdens index maximum of 0.269 and a cut-off value 1.55cm. Hence, when the maximum diameter of the parathyroid nodule was less than 1.55cm, the sensitivity and specificity of residual parathyroid sign in predicting parathyroid adenoma were 63.0% and 63.9%, respectively (Fig.3). A total of 62 of the 107 parathyroid nodules showed signs of polar vascularity. The AUC for predicting polar vascular sign by the maximum diameter of parathyroid nodules was 0.675, significantly different from that of 0.05 (P=0.001). However, the accuracy of using the maximum diameter of parathyroid nodules to predict polar vascular sign is low (0.5<Az0.7), with a Youdens index maximum of 0.346 and a cut-off value of 1.35cm. Hence, when the maximum diameter of the parathyroid nodules is more than 1.35cm, the sensitivity and specificity of polar donor vessel sign in predicting parathyroid adenoma were 79.0% and 55.6%, respectively (Fig.4). When the two signs were displayed at the same time, the joint sensitivity and specificity were 92.2% and 35.5%, respectively.

For the 108 parathyroid nodules in 97 patients with PHPT (of the 107 patients, 10 patients with 12 nodules were excluded for the reason that Tc-99m MIBI SPECT/CT was done in other hospitals or was not done before operation), the sensitivity and accuracy of US for locating the parathyroid nodules were significantly higher than those of Tc-99m MIBI SPECT/CT (93.0% vs. 63.0% and 88.0% vs. 63.0%,respectively; ^2^=26.224, *P*<0.001 for sensitivity; ^2^=18.227, *P*<0.001 for accuracy). The differences between US+Tc-99m MIBI SPECT/CT and Tc-99m MIBI SPECT/CT-alone were statistically significant (^2^=33.410, 21.587, respectively, P<0.001), yet there were no significant differences of sensitivity (^2^=0.866, P=0.352) and accuracy (^2^=0.187, P=0.665) when compared US+Tc-99m MIBI SPECT/CT with US-alone. There were no statistically significant differences in specificity between the three methods (^2^=4.875, *P*=0.087) (Table 2).

**Table 2:** Comparison of sensitivity, specificity, and accuracy in the detection of PHPT via three methods (%)

	Sensitivity	Specificity	Accuracy
US	93.0 (93/100)	25.0 (2/8)	88.0 (95/108)
Tc-99m MIBI SPECT/CT	63.0 (63/100)^a^	62.5 (5/8)	63.0 (68/108)^a^
US+Tc-99m MIBI SPECT/CT	96.0 (96/100)^bc^	12.5 (1/8)	97.0 (97/108)^bc^
^2^	49.554	4.875	30.648
*P*	<0.001	0.087	<0.001

There were significant differences in sensitivity (^2^=49.554, *P*<0.001) and accuracy (^2^=30.648, *P*<0.001) among the three groups, and pairwise comparison was performed for row x column segmentation

^a^Compared with US, the differences of sensitivity (^2^=26.224, *P*<0.001) and accuracy (^2^=18.227, *P*<0.001) were statistically significant

^b^Compared with Tc-99m MIBI SPECT/CT, the differences were statistically significant (^2^=33.410, *P*<0.001 for sensitivity; ^2^=21.587, *P*<0.001 for accuracy)

^c^Compared with US, there were no significant differences (*P*=0.352 and *P*=0.665, respectively). There were no statistically significant differences in specificity between the three methods (^2^=4.875, *P*=0.087)

A total of 93 in 100 (sensitivity=93%) parathyroid lesions were detected by US, while 63 nodules were detected by Tc-99m MIBI SPECT/CT (sensitivity=63.0%). Among the 108 lesions included in US, six cases were false positives (two cases were follicular adenoma, one case was PTMC, one case was nodule goiter, one case was a normal parathyroid gland, and one case was a cervical lymph node), while seven cases were false negative (two cases of the anterior mediastinum, two cases of the superior mediastinum, one case of the posterior pharynx wall, one case of the pericardium, and one case of the superior parathyroid gland). Three cases were false positives in Tc-99m MIBI SPECT/CT, and 37 of 108 lesions were false negatives in Tc-99m MIBI SPECT/CT but positive in US. In addition, ten malignant thyroid nodules were accidentally found in eight patients with thyroid papillary microcarcinomas during the parathyroid US, yet Tc-99m MIBI SPECT/CT could not help in these situations.

There was one patient with a recurrent adenoma after ablation and one patient with in situ recurrence after parathyroid carcinoma, with diameters of 1.3cm and 0.8cm, respectively. US could be used to accurately diagnose these patients, but Tc-99m MIBI SPECT/CT had not detected these two abnormal lesions. There were twelve cases of ectopic parathyroid nodules for which the detection rate of Tc-99m MIBI SPECT/CT was better than that of US (8/12 vs, 6/12, 66.6% vs. 50.0%), including six cases in the ectopic mediastinum, one case in the pericardium, and one case in the posterior pharyngeal wall. In addition, there were four cases of ectopic parathyroid adenoma (intrathyroid parathyroid adenomas) located in the thyroid parenchyma diagnosed by confirmed typical polar vascular sign, while Tc-99m MIBI SPECT/CT identified two in these four cases. Three cases of parathyroid cysts were found by US but not by Tc-99m MIBI SPECT/CT.

## Discussion

Once the diagnosis of PHPT has been established, the detection and localization of the abnormal glands become top priority. Due to the characteristics of embryonic development, the position of the parathyroid gland varies, and the relationship between the parathyroid gland with the recurrent laryngeal nerve and inferior thyroid artery is complicated [9]. Parathyroid nodules often need to be differentiated from thyroid nodules, surrounding soft tissues, and posterior cervical lymph nodes. In this study, two cases of follicular tumors and one case of nodular goiter were mistaken for parathyroid lesions by US because they were located on the dorsal side of the thyroid gland and showed exogenous growth. A case of papillary carcinoma was misdiagnosed as an intrathyroidal parathyroid adenoma by US because of the appearance of polar vascular signs. Tc-99m MIBI SPECT/CT has been considered as the initial diagnostic choice, as it shows both cervical and mediastinal lesions, with high positive predictive value more than 80% [10, 11]. Although the reported sensitivity of Tc-99m MIBI SPECT/CT is high, it may not be sufficient for preoperative localization of parathyroid adenomas when used alone [12, 13].

In this study, the detection rate of abnormal parathyroid by US was significantly higher than ^99m^Tc MIBI SPECT/CT (93.0% vs. 63.0%). In comparison, the sensitivity and accuracy of US in locating parathyroid nodules in 97 cases of PHPT were significantly higher than those of Tc-99m MIBI SPECT/CT (93.0% vs. 63.0% and 88.0% vs. 63.0%). However, a recent meta-analysis included 188 articles and 12 selected original articles with eligibility criteria showed that the pooled sensitivity of the two methods was not statistically different (83% vs. 80%). However, the pooled estimate of 99mTc MIBI SPECT/CT specificity was significantly higher than US [14]. These findings were not consistent with the results in the current study. However, the findings have been conflicting as many studies still demonstrate the superiority of US. There were 37 false negatives in Tc-99m MIBI SPECT/CT and the pathologies were parathyroid adenomas, hyperplasia, and parathyroid cysts, respectively. False negatives in Tc-99m MIBI SPECT/CT is mainly associated with smaller adenomas. In a recent study, Jones et al. [15] showed that Tc-99m MIBI SPECT/CT had a sensitivity of 93% for adenomas>500mg, yet the sensitivity was significantly reduced (51%) for adenomas<500mg. In this study, the maximum diameter of abnormal nodules less than 1cm was detected in 16 cases, accounting for 16.7% of the total 120 nodules. The cellular composition of abnormal parathyroid nodules is also considered to be a factor affecting the accuracy of Tc-99m MIBI SPECT/CT. In research involving 122 cases of parathyroid adenoma, 26 of 68 adenomas (38%) constituted predominantly of chief cells were false negatives on Tc-99m MIBI SPECT/CT, while two (9.0%) of 23 adenomas formed predominantly by oxyphil cells and eight (25.8%) of 31 mixed adenomas were also false negatives on Tc-99m MIBI SPECT/CT [16]. These results suggest that the histological characteristics of parathyroid adenoma influence the imaging results of Tc-99m MIBI SPECT/CT. Although MIBI uptake is not related directly to PTH production and secretion, it is a marker of cell metabolism (mitochondrial uptake). Other factors may also affect the detection of abnormal parathyroid lesions, including serum calcium levels and the cystic degeneration of nodules. Another important confounding factor affecting the diagnostic value of Tc-99m MIBI SPECT/CT is concomitant thyroid nodules, which is known to be more frequent in PHPT patients than in general population. Particularly, some thyroid nodules exhibit intense MIBI uptake early with no tendency to wash-out or delayed wash-out, thereby mimicking parathyroid adenomas. In this study, three cases were false positives for Tc-99m MIBI SPECT/CT, of which the pathologies were nodular goiter, follicular adenoma, and normal parathyroid gland. Since Tc-99m MIBI SPECT/CT is a non-specific tumor imaging agent, it also accumulates in thyroid cancer and thyroid adenomas.

In this study, we also identified that residual parathyroid sign and polar vascular sign are characteristic US manifestations of parathyroid adenomas and this study proposes for the first time that the appearance of these two signs are closely related to the size of parathyroid tumors. The parathyroid gland is mainly composed of a large number of chief cells, along with some eosinophils and cellmatrix proteins. The chief cells are closely arranged, while the cytoplasm and matrix are rich in adipose cells. Hence, US demonstrates homogeneous hyperechoic parenchyma [17].Residual parathyroid signs are presented with a hyperechoic rim or cap around parathyroid hypoechoic tumors on US imaging, which has been confirmed by histopathologic results (Fig.1). Only a few scholars have reported this manifestation of US previously [18].The sensitivity of this sign in the diagnosis of parathyroid nodules is 63.6%, and it is highly sensitive to parathyroid adenomas with a maximum diameter of1.55cm. However, it is difficult to find residual normal parathyroid glands when the tumor is large, which may be caused by smaller residual normal gland tissue and compression. Some pathologists have also proposed that the residual normal parathyroid tissue is a reliable standard for parathyroid adenoma, but it can only be seen in 5060% of cases [19, 20]. This is similar to the results in this study, and the diagnosis of parathyroid adenoma cannot be ruled out when there is no residual normal parathyroid tissue. In this study, three cases of functional parathyroid cysts presented with residual parathyroid signs detected by US but not by Tc-99m MIBI SPECT/CT. Parathyroid cysts are rare and usually occur in the neck of the lower parathyroid gland with high levels of PTH. However, most scholars believe that parathyroid cysts represent degenerative adenomas [21, 22]. In fact, some of these cases in the current study presented with hyperparathyroidism. Approximately 95% of adenomas occur in the neck and usually obtain their blood supply from branches of the inferior thyroid artery. The normal parathyroid gland has no visible flow signal on color and power doppler due to thin nutrient vessels or slow velocity. Parathyroid adenomas are hypervascular and suspended by a vascular pedicle consisting of an extrathyroidal feeding artery enveloped in fat. The polar vascular sign is defined as the presence of an enlarged vascular pedicle around the nodule, which originates from the inferior or superior thyroid arteries (Fig.2). The superior or inferior thyroid artery that terminates at the parathyroid adenoma is thicker than the contralateral artery at a similar position. In this study, the sensitivity of this sign in the diagnosis of parathyroid nodules is 79.0% for parathyroid adenomas with maximum diameter>1.35cm. To our current knowledge, some scholars have reported polar vascular sign but not associated it with the size of abnormal lesions detected by US. Previously, Lane et al. [23] used US to reveal extrathyroidal feeding arteries in the detection of abnormal parathyroid glands, but there were only few cases, no tracking of the whole source of the supply vessels, and no connection between this sign and the size of the parathyroid tumors mentioned. The weight of resected nodule was measured after operation in this literature, but the weight may include tumor and normal parathyroid. Thus, this method may be not objective enough and limits the accuracy of measurement. Meanwhile, some of the postoperative tissues may be broken and could not be weighed precisely. In another study [24], similar findings from 4D CT with polar vascularity were found. However, it is difficult to fully display the whole feeding vessel and their origin. Compared with traditional CT, the ionizing radiation of 4D CT is much higher, and the vascular reconstruction is time-consuming.

We found PTC in ten (10.3%) of the 97 PHPT cases, and 9 out of 10 cases (90%) were PTMC and did not involve any lymph node or distant metastases. The association between thyroid disease and PHPT was first described more than 70years ago [25]. Several reports have described an increased incidence risk of cancer in patients with PHPT [26, 27]. Some scholars have found that PHPT is mainly accompanied by non-medullary carcinomas, especially PTC [28]. Previous studies have uncovered several susceptible factors of PTC in PHPT, such as the tumor-promoting effects of PTH, the goitrogenic effect, and increased mitotic activity induced by hypercalcemia and neck irradiation [29].

There were twelve cases of ectopic parathyroid nodules in this group. For which the display rate of Tc-99m MIBI SPECT/CT was better than that of US (66.6% vs. 50.0%). However, neither of the imaging modalities identified one case located in the posterior pharyngeal wall that was resected by intraoperative PTH monitoring. Tc-99m MIBI SPECT/CT is superior to US in the preoperative display of ectopic parathyroid nodules, especially those of ectopic locations like the retrotracheal region, retrosternal region, upper mediastinal, and intrathymic regions.

FNA is a feasible option for the suspicious nodules that cannot be identified by Tc-99m MIBI SPECT/CT or US. US-guided FNA of the suspected lesion with subsequent PTH measurement in the washout was first applied in the 1980s [30], with a sensitivity of 70100% and specificity of 75100% for parathyroid adenomas [31]. Other scholars believed that FNA has the risk of implantation metastasis along the biopsy tract, which is theoretically possible. However, the true incidence of this complication is controversial. In a study of 81 parathyroid samples, there were no cases of parathyroid tumor metastasis induced by FNA during a follow-up period of 5.8years [32]. Another pitfall of parathyroid biopsy is atypical features, such as capsule infiltration, cellular pleomorphism, fibrous band, and increased proliferation index after the parathyroid biopsy. These histological changes are similar to atypical adenomas or parathyroid carcinomas and may also mislead pathologists [33].

Several limitations should be mentioned for this study. First, because this was a retrospective study, it may have some bias and sampling variation. Secondly, the diagnosis was made by experts in different departments, which may affect the statistical results. Lastly, a small number of negative cases resulted in a low specificity, which may lead to a certain deviation in the results.

## Conclusion

US is highly sensitive, inexpensive, and non-radioactive. However, the accuracy of US is operator-dependent and needs a skillful eye to pick up abnormal parathyroid lesions. The residual parathyroid sign and polar vascular sign are characteristic US features of parathyroid adenomas causing primary hyperthyroidism and the appearance of these two signs is closely related to the size of nodules. US showed excellent diagnostic value for parathyroid adenomas, especially for those Tc-99m MIBI SPECT/CT-negative cases. However, Tc-99m MIBI SPECT/CT is superior to US in the detection of ectopic parathyroid adenomas. In this paper, the combination of US with Tc-99m MIBI SPECT/CT offered the best sensitivity and accuracy in the localization of abnormal glands. Hence, the combined imaging approach may improve surgical outcomes in patients with PHPT.

## Data Availability

Data and materials during the current study are available from the corresponding author upon reasonable request.

## Abbreviations

US: Ultrasonography
PHPT: Primary hyperparathyroidism
PTH: Parathyroid hormone
PTMC: Papillary thyroid microcarcinoma
